# Increasing Partner Attendance in Antenatal Care and HIV Testing Services: Comparable Outcomes Using Written versus Verbal Invitations in an Urban Facility-Based Controlled Intervention Trial in Mbeya, Tanzania

**DOI:** 10.1371/journal.pone.0152734

**Published:** 2016-04-04

**Authors:** Stefanie Theuring, Laura F. Jefferys, Philo Nchimbi, Paulina Mbezi, Julius Sewangi

**Affiliations:** 1 Institute of Tropical Medicine and International Health, Charité—Universitätsmedizin, Berlin, Germany; 2 PMTCT Program Mbeya Region, Ministry of Health and Social Welfare, Mbeya, Tanzania; 3 Regional AIDS Control Program Mbeya, Ministry of Health and Social Welfare, Mbeya, Tanzania; University of Rome Tor Vergata, ITALY

## Abstract

In many Sub-Saharan African settings male partner involvement in antenatal care (ANC) remains low, although great benefits for maternal and infant health outcomes have been long recognised, in particular regarding the prevention of HIV transmission. Yet there is paucity on evidence regarding the effectiveness of strategies to increase male partner involvement. This controlled intervention trial in Ruanda Health Centre in Mbeya, Tanzania, assessed the effectiveness of invitation letters for male involvement in ANC. Pregnant women approaching ANC without partners received official letters inviting the partner to attend ANC. A control group was instructed to verbally invite partners. Partner attendance was recorded at two subsequent ANC visits. Rates for male partner return, couple voluntary counselling and testing (CVCT), and influencing factors were analysed. From 199 ANC clients in total, 97 were assigned to the invitation letter group; 30 of these (30.9%) returned with their male partners for ANC. In the control group of 102 women, 28 (27.5%) returned with their partner. In both groups CVCT rates among jointly returning couples were 100%. Partner return/CVCT rate was not statistically different in intervention and control group (OR 1.2, p = 0.59). Former partner attendance at ANC during a previous pregnancy was the only factor found to be significantly linked with partner return (p = 0.03). Our study demonstrates that rather simple measures to increase male partner attendance in ANC and CVCT can be effective, with written and verbal invitations having comparable outcomes. In terms of practicability in Sub-Saharan African settings, we recommend systematic coaching of ANC clients on how to verbally invite male partners in the first instance, followed by written invitation letters for partners in case of their non-attendance. Further studies covering both urban and rural settings will be more informative for effective translation into policy.

## Background

Throughout Sub-Saharan Africa (SSA), reproductive healthcare systems including services for antenatal care (ANC), voluntary HIV counselling and testing (VCT), or prevention of mother-to-child-transmission of HIV (PMTCT), have widely been established in the past years. Yet they often remain underutilized or suffer from high client attrition rates [1, 2]. At the same time, as an inherent structural deficit of these services, they mostly focus on women and forget the other “half of the equation” of reproduction [3].

It has been shown in multiple studies from SSA that men can positively influence female health decision-making and utilization of reproductive health services, for example the uptake of HIV testing and PMTCT measures [3–7]. The involvement of men in maternal and family health as well as in HIV prevention and PMTCT could significantly contribute to achieving important goals for global public health, particularly Millennium Development Goals 3, 4, 5 and 6, and has been recognized as a priority area of intervention by the World Health Organization [1].

For the United Republic of Tanzania, HIV has been a major public health issue for the past decades, albeit having achieved a reduction of the nationwide prevalence rate from 7% in 2003 to 5.3% in 2012. [8, 9] However great regional differences occur, e.g. in the southwestern Mbeya Region where HIV prevalence amounts to 9%. Mother-to-child-transmission is the second most common way of HIV infection in Tanzania, and at the same time many Tanzanians do not know their HIV status: as of 2012, there was a lack of VCT service utilization of 53% in men and 38% in women in the age group 15–49. [9]

The failure of male participation in ANC and PMTCT has been identified as a key barrier for successful PMTCT measures in Tanzania in the National Strategic Framework for HIV/AIDS. The Ministry of Health and Social Welfare had therefore proposed a target to increase male partner testing during pregnancy to 50% by the year 2015. [8]

As an effective tool to systematically increase partner involvement, official partner invitation letters have been frequently mentioned in studies from other African settings. [5, 10, 11] Although research from Mbeya Region revealed several years ago that men considered official invitations both helpful and acceptable [12, 13], this specific strategy has not, to date, been further assessed regarding its effectiveness in Tanzania. Therefore, this study aimed at analysing partner return rates and couples´ VCT (CVCT) rates following official partner invitation letters compared to verbal invitations in Mbeya, Tanzania.

## Methods

### Study design

Within the frame of a multi-site implementation study [13] regarding acceptability of invitation letters for partner involvement in Mbeya Region, Tanzania, a facility-based controlled intervention trial was conducted as a sub-study to assess the measure´s effectiveness in Ruanda Health Centre (HC) in Mbeya. Ruanda HC was chosen for the study as it is a large, urban-situated primary healthcare facility offering free-of-charge ANC, VCT, PMTCT and HIV treatment services, and serves up to 300 new ANC clients monthly. Prior to the study period, few male partners joined their pregnant wives for CVCT. Available data from the clinic registers indicated that over the study-preceding three-month period, only 1.7% of partners of all new ANC clients at Ruanda HC had received CVCT. Standard ANC services at Ruanda HC include monthly visits to the facility; with routine opt-out HIV testing offered to women at their initial ANC visit.

Women attending ANC for the first time during their current pregnancy were enrolled for the study within a four-week-period between April and May 2013. Eligibility criteria included confirmed pregnancy, written informed consent, and a partner who was generally accessible (i.e., not permanently working abroad). Women were excluded if they were widowed or divorced or if their partner attended the first ANC visit. After enrolment, women were allocated to two different groups in a quasi-randomised approach by systematic alternation of intervention and control group; i.e. the first client was allocated to intervention, the second to control, the third to intervention, and so forth.

### Study procedure

Interviews with women were conducted in Swahili by trained research assistants at baseline visit and at two subsequent ANC visits, and were based on structured questionnaires which had been pre-tested and adjusted accordingly by the investigators. Questionnaires were similar for both groups apart from questions directly targeting the invitation letter or verbal invitation. Interviews were conducted after routine ANC sessions according to the facility´s standard of care (including opt-out HIV testing) in a separate room and not in the presence of the ANC nursing staff or the male partner.

After the first ANC visit, women were interviewed on baseline socio-demographic indices about themselves and their partners. “Employment” included formal and self-employment as opposed to “unemployment”. “Media exposure” was grouped in having home access to not more than one media type, including radio, television or newspaper, or having access to two or all of these media types. “Intimate partner violence” was defined as the experience of financial, emotional or physical abuse from the partner. Results of HIV tests performed within the study sites were not known to the research team, therefore data on participant and partner HIV status were self-reported by participants. Because very few women in this study reported to be HIV-positive or to have an HIV-positive partner, we assumed some degree of underreporting and included only unspecified “HIV status knowledge” as a baseline variable.

At the end of the first interview women of the intervention group were given a written invitation letter for their male partner, requesting their presence at the next routine ANC visit. It contained general information about what to expect during an ANC session, but did not state that an HIV test would be offered. The courteous and formal letter contained the partner´s name and was signed by the Regional Medical Officer. If women reported their partner to be illiterate, they were requested to identify a person who would read the letter to the partner. Women in the intervention group did not receive additional instructions to verbally invite their partner.

Within the control group, women did not receive a letter, but were instructed to verbally invite their male partners to attend the next ANC session. Both groups received a specified appointment for the next ANC visit in 4 weeks.

If the partner attended the next visit as requested a joint ANC session would take place during which CVCT was offered to the couple. If either of the couple did not want to take part in CVCT, VCT was offered individually. After this joint session the research assistant would interview the woman on the partner session.

If the partner had not accompanied the woman to this second ANC visit, they were interviewed regarding the reasons for his non-attendance. These women then received either a further letter of invitation for the partner, or, if belonging to the control group, repeated instructions to verbally invite the partner to the next ANC session. Again, both groups received a new specified appointment in 4 weeks.

After the third ANC visit women of both groups were again interviewed by the research assistants regarding partner return at this third visit, CVCT, or reasons for the partner to reject attending ANC.

During the study period, irregular community sensitisation on HIV testing and PMTCT took place as part of routine government public health initiatives in Mbeya Region, for example in the form of radio broadcasts, but not specifically in Ruanda HC.

### Statistical methods

Data analysis was performed using IBM SPSS Statistics for Windows, Version 22.0. Primary outcome indicators were partner return rate and CVCT rate; analysis of these indicators was based on intention-to-treat. Secondary outcome indicators were influencing socio-demographic factors on partner return and CVCT. Intervention and control group were compared regarding socio-demographic indices using Pearson´s Chi² Test and Fisher´s Exact Test for categorical variables, and using Mann-Whitney-U Test for continuous variables. Influencing factors on partner return and CVCT were assessed by calculating univariate odds ratios (OR) of relevant variables. Multivariable analysis of factors associated with male partner involvement was conducted using a binary logistic regression model, and included the variables that had a p-level<0.20 [14] in univariate analysis as well as significantly imbalanced baseline covariates and the intervention group type to adjust for potential selection bias despite randomization. A significance level of p<0.05 and a 95% confidence interval (CI) were used throughout.

### Ethical considerations

The study was approved by the Mbeya Medical Research and Ethics Committee in Tanzania (MRH/R10/18/VOL.V1/75). All participants gave written informed consent and could withdraw their participation at any time without stating reasons. The research assistants´ training included participant confidentiality, and all data was recorded and treated anonymously.

## Results

**Fig 1:**
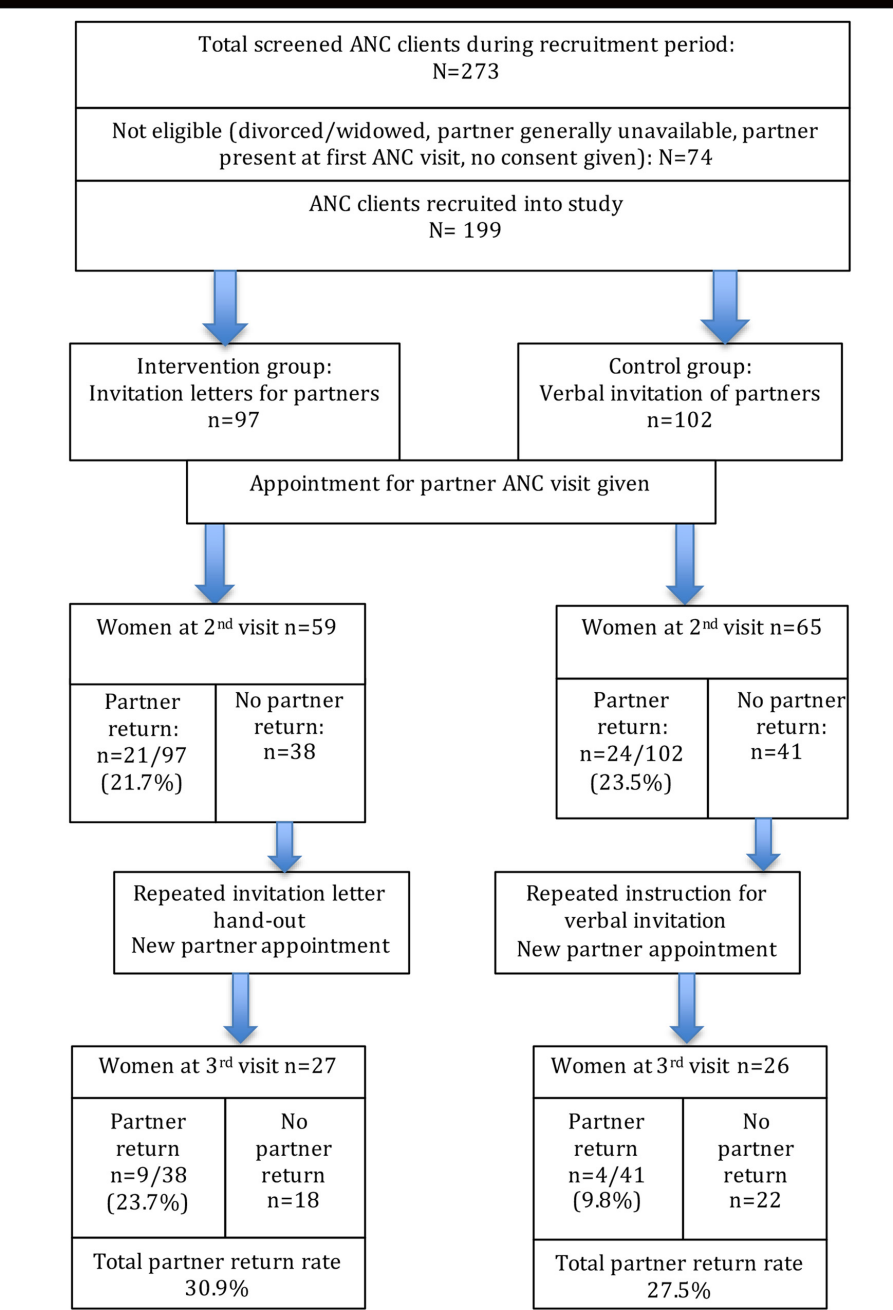
Flow chart: Study participants and partner return rates at each visit.

The study cohort consisted of 199 eligible women; 97 women were part of the intervention group and 102 were part of the control group (Fig 1).

In the intervention group the study participants´ response rate for a second ANC visit was 60.8% (59/97), in the control group it was 63.7% (65/102). From the women who had not brought their partner at the second visit and were given a new partner appointment for the third visit, 71.1% (27/38) of women from the intervention group and 63.4% (26/41) from the control group returned.

Being lost to follow-up after the first or after the second visit was not associated with advanced gestational age at the first ANC visit nor with any other socio-demographic maternal or partner factors apart from maternal age (response rate for 2^nd^ visit if <21 years: 75% vs 58.4%, p = 0.046) and employment (response rate for 2^nd^ visit if employed: 67.6% vs. 46%, p = 0.007).

The women had a median age of 24 years (range 18–44 years) and presented to ANC at a median gestational age of 19 weeks (range 5–35). The majority (73.8%) were living in informal relationships, without being married. Socio-demographic indices did not differ between intervention and control group except for literacy and HIV status knowledge (Table 1). HIV status knowledge was significantly linked with secondary school education in both women (p = 0.048) and partners (p = 0.014).

**Table 1:** Socio-demographic baseline information and differences between intervention and control group.

Characteristics of Study Participants (n = 199)	All	Intervention group (n = 97)	Control group (n = 102)
**Age** *n*	**198**		
*Median (range)*	24 yrs (18–44)	24 yrs (18–44)	24 yrs (18–25)
**Literacy** *n (%)*	**198**		
Literate	190 (96.0)	90 (92.8)	100 (99.0)*
Illiterate	8 (4.0)	7 (7.2)	1 (1.0)
**Religion** *n (%)*	**197**		
Christian	184 (93.4)	91 (94.8)	93 (92.1)
Muslim & Other	13 (6.6)	5 (5.2)	8 (7.9)
**Education** *n (%)*	**198**		
Up to primary	130 (65.7)	67 (69.1)	63 (62.4)
At least secondary	68 (34.3)	30 (30.9)	38 (37.6)
**Employment** *n (%*)	**198**		
Employed	148 (74.7)	75 (77.3)	73 (72.3)
Unemployed	50 (25.3)	22 (22.7)	28 (27.7)
**Home media exposure** *n (%)*	**199**		
Up to 1 media type	117 (58.8)	57 (58.8)	60 (58.8)
2 media types or more	82 (41.2)	40 (41.2)	42 (41.2)
**HIV status knowledge** *n (%)*	**198**		
Known status	91 (46.0)	37 (38.1)	54 (53.5)**
Unknown status	107 (54.0)	60 (61.9)	47 (46.5)
**Gestation** *n*			
*Median (range)*	19 wks (5–35)	20 wks (7–34)	19 wks (5–35)
**Primigravidae**	**199**		
Yes	95 (47.7)	44 (45.4)	51 (50.0)
No	104 (52.3)	53 (54.6)	51 (50.0)
**Travel time to clinic** *Median (range)*	30 min (5–360)	30 min (5–360)	30 min (5–90)
**Travel cost to clinic** *Median (range)*	800 TSH (0–4000)	800 TSH (0–4000)	800 TSH (0–3000)
**Marital status** n (%)	**198**		
Married	52 (26.3)	28 (28.9)	24 (23.8)
Informal partnership	146 (73.8)	69 (71.1)	77 (76.2)
**Partnership duration** *n*	**197**		
Median (range)	3 yrs (0–25)	4 yrs (0–17)	2 yrs (0.25–25)
**Partner in previous ANC** *n*	**104**		
No	72 (69.2)	36 (67.9)	36 (70.6)
Yes	32 (30.8)	17 (32.1)	15 (29.4)
**Ever jointly HIV tested** n (%)	**190**		
No	142 (74.7)	74 (79.6)	68 (70.1)
Yes	48 (25.3)	19 (20.4)	29 (29.9)
**Partner´s age** *Median (range)*	29 yrs (20–52)	29 yrs (20–49)	29.5 yrs (20–52)
**Couple age difference** *Median (range)*	5 yrs (-10–20)	4 yrs (-10–20)	5 yrs (-1–19)
**Partner literacy** *n (%)*	**199**		
No	2 (1.0)	1 (1.0)	1 (1.0)
Yes	197 (99.0)	96 (99.0)	101 (99.0)
**Partner education** *n (%)*	**198**		
Up to Primary	101 (51.0)	51 (53.1)	50 (49.1)
At least Secondary	97 (49.0)	45 (46.9)	52 (51.0)
**Partner employment** *n (%)*	**198**		
Employed	185 (93.4)	88 (91.7)	97 (95.1)
Unemployed	13 (6.6)	8 (8.3)	5 (4.9)
**Partner status known** *n (%)*	**167**		
Not known	132 (66.3)	69 (71.1)	63 (61.8)
Known	67 (33.7)	28 (28.9)	39 (38.3)
**Intimate partner violence** *n (%)*	**197**		
No	123 (62.4)	60 (63.2)	63 (61.8)
Yes	74 (37.6)	35 (36.8)	39 (38.2)

*Fisher´s Exact Test p = 0.03

**Pearson´s Chi^2^ Test p = 0.03

All other variables did not differ on a significant level

### Partner return and CVCT rates

In the intervention group 59 women attended the 2^nd^ visit, of which 56 (94.2%) reported that they had handed out the letter to their partners at home. Twenty-one men accompanied their partners to the 2^nd^ ANC appointment as requested. Nine partners returned at the third visit after having received a 2^nd^ invitation, hence intention-to-treat-analysis resulted in a total of 30 out of 97 (30.9%) partners attending ANC in the intervention group.

In the control group 65 women attended the 2^nd^ visit, of which 62 (93.9%) reported that they had verbally invited their partner for the next ANC visit as instructed. Twenty-four returned with their partner to the 2^nd^ ANC visit. Including an additional 4 partners who returned at the 3^rd^ visit, a total of 28 out of 102 (27.5%) partners attended ANC in the control group. Odds for partner attendance following invitation letters were increased by 20% compared to verbal invitations (OR 1.2; 95% CI 0.6–2.2), although no statistically significant difference was found between intervention and control group for this primary outcome indicator. The absolute risk difference between the two groups was a 3.4% higher partner return rate in the written invitation group.

Partner return rates were similar at the 2^nd^ ANC visit in intervention and control group (21.7% vs 23.5%). However, based on intention-to-treat among women without partner attendance at the 2^nd^ visit, there was an almost triplicate partner return rate at the 3^rd^ ANC visit in the intervention group compared to control (23.7% vs 9.8%, OR 2.9, CI 0.8–10.3). The main reason for non-attendance at the second visit was partners´ job-related unavailability, which was stated by 42/78 (53.9%) women with non-returned partners of both groups. Travel for family reasons was mentioned by 10/78 (12.8%) women, while unwillingness of the partner to attend was reported by 9/78 (11.5%) of women.

All of the 58 attending partners in either the intervention or the control group accepted CVCT. Test results from CVCT differed between the groups: while in the intervention group, CVCT revealed a 20.7% HIV prevalence in women and a 26.7% HIV prevalence in partners, in the control group none of the jointly tested women or men reported to be HIV positive (Fisher´s exact test p = 0.027 and 0.005 respectively). Among HIV-affected couples from the intervention group, four jointly tested couples were serodiscordant, in three cases the man being infected, and five couples were concordantly HIV-positive.

### Factors influencing partner return and CVCT

Looking for socio-demographic influencing factors in univariate analysis, we found only ANC attendance of the partner in a previous pregnancy to be significantly associated with partner return and CVCT (p = 0.03). All other characteristics of the women or partners did not reveal any association of statistical significance (Table 2). Women´s literacy triplicated the odds of partner return, but showed no statistical significance, probably due to the overall low numbers of illiterate women in the study. In multivariate analysis, the factor of previous partner ANC attendance remained a significant predictor of partner return and CVCT when controlling for intervention group, women´s literacy, and baseline HIV status knowledge (AOR 3.2, CI 1.2–8.1, p = 0.02; Table 2).

**Table 2:** Univariate and multivariate analysis of influencing factors on partner attendance/CVCT.

Study participants´ characteristics at baseline	n	Partner attendance/ CVCT	Unadjusted OR (95% CI)	p	Adjusted OR (95% CI)	p
**Assigned Group**	**199**			0.59		0.50
Intervention	97	30.9%	1.2 (0.6–2.2)		1.4 (0.6–3.3)	
Control	102	27.5%	1.0		1.0	
**Age** *Median (range)*	24 yrs (18–44)	24 yrs (18–37)		0.85^§^		
**Literacy** *n*	**198**			0.44*		0.56
Literate	190	30.0%	3.0 (0.4–25)		2.0(0.2–21.2)	
Illiterate	8	12.5%	1.0		1.0	
**Religion**	**197**			0.76		
Christian	184	29.9%	1.0			
Muslim & Other	13	23%	0.7 (0.2–2.7)			
**Education**	**198**			0.40		
Up to primary	130	30.8%	1.0			
At least secondary	68	25.0%	0.8 (0.4–1.5)			
**Employment**	**198**			0.34		
Unemployed	50	24.0%	1.0			
Employed	148	31.1%	1.4 (0.7–3.0)			
**Home media exposure**	**199**			0.98		
Up to 1 media type	117	29.1%	1.0			
2 media types or more	82	29.3%	1.0 (0.5–1.9)			
**Self-reported HIV status**	**198**			0.84		0.71
Known status	91	28.6%	1.0		1.0	
Unknown status	107	29.9%	1.1 (0.6–2.0)		0.8 (0.3–2.1)	
**Gestation** *Median (range)*	19 wks (5–35)	18 wks (7–34)		0.89^§^		
**Primigravidae**	**199**			0.92		
Yes	95	29.5%	1.03(0.6–1.9)			
No	104	28.9%	1.0			
**Travel time to clinic** *Median (range)*	30 min (5–360)	25 min (5–360)		0.56^§^		
**Travel cost** *Median (range)*	800TSH (0–4000)	800TSH (0–3000)		0.73^§^		
**Partnership type**	**198**			0.79		
Married	52	30.8%	1.0			
Informal partnership	146	28.8%	0.9 (0.5–1.8)			
**Partnership duration** *Median (range)*	3 yrs (0–25)	3 yrs (0.25–18)		0.79^§^		
**Partner in previous ANC**	**104**			**0.03**		**0.03**
No	72	22.2%	1.0		1.0	
Yes	32	43.8%	2.7 (1.1–6.7)		2.8 (1.1–7-0)	
**Ever jointly HIV tested**	**190**			0.54		
No	142	29.6%	1.0			
Yes	48	25.0%	0.8 (0.4–1.7)			
**Partner´s age** *Median (range)*	29 yrs (20–52)	30 yrs (20–51)		0.78^$^		
**Couple age difference** *median (range)*	5 yrs (-10–20)	5 yrs (-2–17)		0.80^$^		
**Partner literacy**	**199**			1.0*		
Yes	197	29.4%				
No	2	0%				
**Partner education**	**198**			0.86		
Up to Primary	101	29.7%	1.0			
At least Secondary	97	29.9%	1.1 (0.6–2.0)			
**Partner employment**	**198**			0.53*		
Employed	158	28.1%	0.6 (0.2–2.0)			
Unemployed	13	38.5%	1.0			
**Partner status known**	**199**			0.63		
Not known	132	28.0%	0.9 (0.5–1.6)			
Known	67	31.3%	1.0			
**Intimate partner violence**	**197**			0.75		
No	123	27.7%	1.0			
Yes	74	29.7%	1.1 (0.6–2.1)			

*Fisher´s Exact Test

^§^Mann-Whitney-U Test

All other: Pearson´s Chi^2^ Test; Significant p-levels printed in bold

## Discussion

In this study we analysed the effectiveness of official invitation letters in comparison to verbal invitations for increasing male partner attendance at ANC and CVCT. We found that both measures resulted in high partner return rates: almost every third man came to the next ANC visit with his female partner in both groups. Although official invitation letters brought a 20% increased odds of partner return compared to verbal invitations, both measures led to comparable outcomes.

The results of our investigation largely correspond to the findings of other studies on partner return following official invitation letters, e.g. 26%-35% in South Africa [3], 28% in Malawi [15] or 36% in Kenya [16], while some others reported lower rates, for example in a Ugandan study with 16% partner return [11]. In another Tanzanian study, although with a slightly different focus, 33% of women returned with partners [17]. In studies where women were instructed to verbally invite their partner, male attendance at ANC was usually lower, for example in Kenya with 11% [4] or in Tanzania with 12% [7]. However our overall partner return rates are still below the national Tanzanian goal of 50% partner testing during pregnancy [8]. Other authors have reported invitation letters to result in around 50% partner return, and even above 70% when combined with home visits [18]. An earlier study from the same Tanzanian setting [13] demonstrated that invitation letters might have considerably different outcomes according to the hospital setting, with partner return rates as high as 76% in rural areas.

A reason for the surprising finding that both groups resulted in very similar outcomes could be the fact that while women in the control group had received comprehensive coaching on how to verbally invite their partner, women in the intervention group had not received this coaching in addition to the letter. It is possible that personal ambition of the woman to invite the partner compared to only handing out a letter does play a role in finally convincing him to attend. Apart from that, another reason could be that Ruanda HC had extremely low partner attendance rates before study start (1.7%), and no specific measures were in place to involve men in this facility. This might have contributed to the finding that even a relatively basic intervention like being instructed by health staff to verbally invite the partner for the next appointment already led to an impressive gain in partner return. Previous studies from the same setting in Tanzania have found that partner non-attendance is often a result of men lacking information regarding their expected attendance at ANC/PMTCT services [12], and our results imply in fact that as long as the information is passed on to male partners, every means of passing on this information can be highly effective.

Even though invitation letters and verbal invitations resulted in a similar return rate at the second ANC visit, it was observed that in case of a repeated invitation of the partner, official invitation letters had a stronger effect. It is possible that a letter with a formal character is more impressive if handed out repeatedly, and also that women are reluctant to insistently invite partners verbally several times if this was unsuccessful earlier. Additionally, official letters seemed to have an encouraging character specifically for HIV-infected partners. While the invitation letter used in this study did not contain information on HIV testing, and some authors have reported this to be a deterrent from partner attendance [10], other studies have demonstrated that the perspective to receive an HIV test can motivate men to attend ANC, with improved partner attendance rates compared to a letter without information on CVCT. [3, 19] Further implementation research in our setting could reveal if there would be an additional increase in partner return rates if invitation letters mention that HIV testing will be offered.

However, as observed in our study, even official invitation letters not mentioning CVCT seem to provide HIV-infected individuals with more confidence in benefit of attendance compared to verbal invitations. Both measures resulted in a notable increase of partner return compared to the pre-intervention data. Considering practicability in resource-constrained settings like SSA, we suggest that at the first instance, ANC clients should receive intensive coaching on verbal partner invitation, which should then be followed by an official invitation letter if partners did not return after verbal invitation.

All ANC-attending partners consented to CVCT, exceeding other studies´ outcomes, where mostly around 90% of partners in ANC proceeded to CVCT. [3, 11] Becker et al. reported even only 47% of letter-invited couples consenting to CVCT in Tanzania [17]. The full CVCT coverage among attending partners suggests that Ruanda HC is providing high quality couple pre-test counselling, and further research, possibly using qualitative methods, would be insightful to assess the successful counselling strategies in this facility.

At study enrolment, half of the women did not know their HIV status and two-thirds did not know their partners´ status. This problem has also been reported from a South African study, where 40% of recently pregnant women did not know their partners´ HIV status even post-delivery [20]. The increased CVCT rate and status awareness in our study setting have to be viewed as a large success in addressing a high priority issue especially in the context of the women´s pregnancies and future PMTCT measures.

We found ANC attendance of the partner in a previous pregnancy to be the only significant predictor for partner return and CVCT. This finding demonstrates that partner attendance in ANC is acceptable for those men who have already participated in it, and that the inherent cultural barrier can be overcome by individual positive experiences. Previous studies in this context have identified a number of other influencing factors, like partners´ secondary education [21], or gestational age [3], but this was not confirmed by our data, or, in the case of maternal HIV status, not analysed due to lacking reliable data. For future research, it would be helpful to include laboratory-confirmed maternal HIV status in the analysis of predictors for partner return.

However, some influencing factors might have remained unidentified due to lacking statistical power of the remaining sample after the second ANC visit. We acknowledge that the rather high drop-out of women after the first visit is a limitation of this study, decreasing the sample size for follow-up data and thereby reducing the power of the trial. Response rate in our cohorts for second ANC visit was about 60%, which is largely in accordance with the regional setting [9]. Drop-out after the first ANC visit has frequently been reported and might be caused by women changing the health facility for ANC, transportation problems, or low risk perception for their pregnancy [11]. For similar studies in the future, the primary estimated sample size should take the possibility of high attrition rates at return visits into consideration, and studies should be sufficiently powered to identify significance in smaller differences between the two study arms than the one our power calculation was based on.

Another limitation of the study was the mode of chronological alternating randomization, which is only a quasi-randomized approach, and although our results do not imply this, we cannot completely exclude a selection bias. Also, we are aware of the fact that a single-site study might not provide generalizable results, and further studies should be conducted, especially comparing outcomes between urban and rural settings.

In conclusion, our study, conducted in an urban setting in Tanzania, demonstrates that rather simple measures to increase partner attendance in ANC and CVCT, including official partner invitation letters and instructions for women to verbally invite their partners, can be effective and have comparable outcomes. Partner involvement in ANC and CVCT is viewed as a paramount factor for improving reproductive, maternal and infant health-related parameters, particularly in the context of HIV and PMTCT. While further studies using larger samples and different geographical settings, covering both urban and rural, would be helpful for evidence-based policy-making, we recommend the systematic coaching of ANC clients on how to invite partners verbally at the first instance, followed by official partner invitation letters if verbal invitations are not successful. These strategies should be embedded as standard procedures in ANC guidelines to increase male partner involvement in those health services.
